# Discovery and Validation of Potential Serum Biomarkers with Pro-Inflammatory and DNA Damage Activities in Ulcerative Colitis: A Comprehensive Untargeted Metabolomic Study

**DOI:** 10.3390/metabo12100997

**Published:** 2022-10-20

**Authors:** Mingxiao Li, Rui Zhang, Mingjie Xin, Yi Xu, Shijia Liu, Boyang Yu, Boli Zhang, Jihua Liu

**Affiliations:** 1 Jiangsu Provincial Key Laboratory for TCM Evaluation and Translational Development, School of Traditional Chinese Pharmacy, China Pharmaceutical University, Nanjing 211198, China; 2 Department of Pharmacy, Jiangsu Province Hospital of Chinese Medicine, Affiliated Hospital of Nanjing University of Chinese Medicine, Nanjing 210029, China; 3 State Key Laboratory of Modern Chinese Medicine, Tianjin University of Traditional Chinese Medicine, Tianjin 301617, China

**Keywords:** pyroglutamic acid, ulcerative colitis, biomarker, inflammation, DNA damage

## Abstract

Ulcerative colitis is a type of non-specific inflammatory bowel disease with unclear etiology. It is considered a progressive disease with risks of bowel motility disorders, anorectal dysfunction, and even colorectal cancer. Commonly used diagnostic markers have poor specificity and cannot predict the development of ulcerative colitis. In this study, 77 serum samples (31 patients, 46 healthy controls) were analyzed using high performance liquid chromatography-quadrupole time-of-flight mass spectrometry and 31 metabolites with significant level changes were found, revealing the relationship of ulcerative colitis to disturbed glutathione metabolism and caffeine metabolism. In addition, pyroglutamic acid, a biomarker of cervical cancer and gastric cancer, was identified with elevated levels in the serum of ulcerative colitis patients. The role of pyroglutamic acid was further analyzed, and the results indicated its positive correlation with the upregulation of inflammatory factors and increased levels of phosphorylated histone H2AX (*γ*H2AX) in IEC-6 cells, which are related to DNA damage. All these results suggest that pyroglutamic acid is not only a biomarker for distinguishing ulcerative colitis status, but that it is also a potential effective metabolite that promotes the transformation of ulcerative colitis to colorectal cancer.

## 1. Introduction

Ulcerative colitis (UC) is a chronic inflammatory disorder of the colonic mucosa, which is initiated in the rectum, that causes superficial damage to the bowel wall and extends proximally to the colon [1,2,3]. The most common clinical symptoms are bloody diarrhea, and the clinical course is alternated by periods of exacerbation and remission [4,5]. UC is now considered a progressive disease with risks of bowel motility disorders, anorectal dysfunction, and even colorectal cancer (CRC) [6]. The overall risk of colorectal cancer is 0.1% in the first decade followed by 2.9%, 6.7%, and 10.0% by the second, third, and fourth decades after UC symptom onset [7,8]. Patients with longer durations of UC should be especially vigilant about the increased risk of CRC.

At present, there is no suitable biomarker to distinguish UC status or predict the progress of the disease. In the development of sensitive and convenient tools for diagnosis and monitoring, various biomarkers have been identified, such as fecal calprotectin or lactoferrin [9,10]. However, these biomarkers are not found exclusively in UC patients, and can also be elevated in other inflammatory intestinal disorders, such as gastrointestinal malignancies, nonsteroidal anti-inflammatory drug (NSAID) enteropathy, and diverticulitis [11,12].

Metabolomics is a new biological system research method developed after genomics and proteomics. Metabolomics technology combines the acquisition of multiparameter metabolic data with multivariate pattern recognition analysis. Through the analysis of all the small molecular substances in the biological liquid and tissue, a comprehensive exploration of metabolic changes and metabolomic networks of human diseases can be revealed [13,14]. Nuclear magnetic resonance (NMR) spectroscopy and mass spectrometry are commonly used as analytical platforms. Unlike targeted metabolomics, which only focuses on specific substances, untargeted metabolomics collects as much information as possible and has a wider coverage of substances. Disturbances found in local and systemic metabolism can not only provide biomarkers for the clinical diagnosis of disease but may also be new targets for the treatment of disease [15,16,17,18].

In this study, we performed an untargeted metabolomic profiling of serum samples of UC patients and healthy controls using HPLC-Q-TOF/MS. Our aim was (1) to investigate possible differences in metabolite levels between UC patients and healthy controls, (2) to screen potential diagnostic biomarkers for UC, and (3) to validate the potential pro-inflammatory and DNA damage activity of these biomarkers. This study presents potential effective biomarkers for clinical diagnosis and progression prediction of UC and provides targets for its clinical treatment.

## 2. Materials and Methods

### 2.1. Population and Study Design

Patients with UC were recruited at the Affiliated Hospital of Nanjing University of Chinese Medicine. Clinical doctors confirmed UC diagnosis based on the results of a medical history, physical examination, colonoscopy, and histopathology. Patients of both sexes, aged between 18 and 65 years, diagnosed with ulcerative colitis, and who agreed to participate in this study were recruited. Controls were matched by age and gender with the UC patients.

Patients with other diseases that affect metabolic spectra and physiological indicators were excluded. The detailed exclusion criteria were: (1) patients with bacillary dysentery, amebic dysentery, chronic schistosomiasis, intestinal tuberculosis, infectious colitis, ischemic enteritis, or radiation enteritis; (2) patients with severe complications such as local stenosis, intestinal obstruction, intestinal perforation, toxic megacolon, massive hemorrhage, colon cancer, or rectal cancer; (3) patients with other primary and secondary infectious diseases, such as cholecystitis and pneumonia; (4) patients with other serious cardiovascular, liver, gallbladder, lung, kidney, or blood system diseases.

Thus, a total of 77 adult individuals, including 31 UC patients and 46 healthy controls, participated in this study. The participants were asked to complete a questionnaire on their medical history and Mayo scores and signed an informed consent form. The clinical and demographic characteristics of the study population are presented in Table S1. Serum samples collected from all participants were stored at −80 °C until analysis.

### 2.2. Preparation of the Samples for Metabolomics Extraction

Serum samples were obtained from whole blood, which were collected in 2.4 mL clotting activator vacuum system tubes. The tubes were stored in the vertical position at room temperature for 60 min to permit the formation of a clot. At the end of the clotting period, blood samples were centrifuged in a horizontal rotor for 10 min at 1300 g at room temperature. Then, serum fractions were collected in Eppendorf tubes and stored at −80 °C until the day of analysis.

An aliquot of 50 μL was precipitated by adding 150 μL of methanol (1:3, *v*/*v*), which was mixed for 60 s. Precipitated protein was subsequently removed by centrifugation (12,000 rpm, 10 min) at 4 °C. Then, the supernatant was transferred to a tube and dried under a gentle stream of nitrogen at room temperature. Finally, the supernatant was reconstituted with 50 μL methanol and filtered through a 0.22 µm nylon filter into glass vials for further analysis. Quality control (QC) samples were prepared by mixing an equal volume of all samples.

### 2.3. HPLC-Q-TOF MS Analysis

Metabolite separation was performed using an Agilent Technologies 6530 Accurate-Mass Q-TOF LC/MS system (Santa Clara, CA, USA). The mobile phase consisted of water and 0.1% formic acid (eluent A) and acetonitrile (eluent B). Serum analyses were archived on an Agilent Zorbax extend C18 column (150 × 4.6 mm i.d., 5 μm). The flow rate was 1.0 mL/min with solvent A (water with 0.1% formic acid) and solvent B (acetonitrile). The chromatographic gradient was started at 5% phase B for the first minute, followed by an increase of phase B to 95% (from 0 to 50 min), and was then kept at 95% for 10 min (from 50 to 60 min). The gradient returned to initial conditions (5% phase B) in 0.5 min, which were maintained for 10 min. The injection volume of all samples was 10 μL. A calibrating solution containing reference masses at *m*/*z* 121.0509 (protonated purine) and *m*/*z* 922.0098 (protonated hexakis [1H,1H,3H-tetrafluoropropoxy]) in positive ion mode was continuously introduced. Mass spectrometry was performed with an electrospray ionization ion source in the positive (ESI+) ion mode. The MS parameters were set as follows: fragmental voltage at 120 V, nebulizer gas at 35 psig, capillary voltage at 4000 V, drying gas flow rate at 9 L/min, and temperature at 325 °C. The data were collected in centroid and profile mode with a mass range of 50–1500 *m*/*z* using the high-resolution mode (4 GHz).

### 2.4. Method Assessment

The repeatability and robustness of the current experiment were tested using pooled quality control samples (QC), which were randomly injected throughout the sequence list. An unsupervised PCA analysis showed good repeatability and robustness of the analytical method (Figure S1). We also evaluated the total ion chromatograms (TICs) of the QC samples (Figure S2), which showed that the response intensity and retention time of each chromatographic peak overlapped. Three different ions in the TICs of the QC samples were extracted for the assessment of method validation. Method repeatability RSD% values of retention times and peak areas of three different ions were calculated from tested QC samples, as shown in Table 1. These data demonstrated a high reproducibility of the method and the stability of the instrument during the experiment.

### 2.5. Data Processing and Statistical Analysis

The raw data acquired from Q-TOF LC/MS ESI+ analysis were transformed to data format (.mzdata) files using MassHunter Workstation Software (Version B.06.00, Agilent Technologies, Santa Clara, CA, USA). We used R Foundation for statistical computing, data pretreatment procedures such as nonlinear retention time alignment, peak discrimination, filtering, alignment, and matching. The ion features present in less than 80% of samples were screened out.

The intensities of each peak detected were generated by virtue of the retention times and the *m*/*z* data pairs for each ion. After being log-transformed, Pareto-scaled, and normalized to peak intensity, the processed data were imported into SIMCA-P 14.1 (Umetrics, Umeå, Sweden) and MetaboAnalyst (https://www.metaboanalyst.ca/) (accessed on 19 June 2021), where they were subjected to multivariate data analyses, including principal component analysis (PCA) and orthogonal partial least-squares discriminant analysis (OPLS-DA). False discovery rate adjusted *p*-values < 0.05 and variable importance in the projection (VIP) values > 1.0 were used to screen for the significantly different metabolites. The online databases HMDB (http://www.hmdb.ca/) (accessed on 6 August 2021), METLIN (http://metlin.scripps.edu/) (accessed on 15 August 2021), and MassBank (http://www.massbank.jp/) (accessed on 22 August 2021) were used to identify the potential metabolites by matching with the structure messages of metabolites. They were selected when the difference between observed and theoretical mass was below 10 ppm. After the *m*/*z* values and isotopic ions were matched, the second-order fragment ions were analyzed to identify metabolites using MassBank.

### 2.6. Cell Culture

IEC-6 rat epithelial cells were purchased from ATCC and cultured in DMEM containing 10% *v*/*v* fetal bovine serum (FBS) at 37 °C in a humidified atmosphere containing 5% CO_2_. For qPCR, Western blot, and comet assay experiments, IEC-6 cells were plated at 4 × 10^6^/well in 6-well tissue culture plates, while for immunofluorescence experiments, IEC-6 cells were plated on coverslips in 24-well plates at 1 × 10^5^/well. Both of these were allowed to adhere overnight and were then cultured with 10, 100, and 1000 μM pyroglutamic acid for 24 h.

### 2.7. Analysis of mRNA Levels by Quantitative Real-Time PCR (qPCR)

The total RNA was extracted using TRIzol reagent (Invitrogen Life Technologies, Carlsbad, CA, USA). The RNA concentration was determined using a spectrophotometer at 260 nm and 280 nm. Equal amounts of RNA (1 μg) were reverse transcribed into cDNA, and the cDNAs were used as templates for PCR amplification. A QuantStudio 3 Real-Time PCR System and fast gene-expression method were used with the following cycling conditions: 95 °C for 5 min, followed by 45 cycles at 95 °C for 10 s, 57 °C for 20 s, and 72 °C for 20 s. Then, melt curve analysis was performed by raising the temperature from 61 °C to 95 °C at a rate of 0.15 °C/s. *β*-actin was used as an internal control to normalize the variability in expression levels. The 2^−ΔΔCT^ (cycle threshold) method was used to calculate the results and the mRNA expression levels are presented as a fold-change compared to control, which was set as 1. The specific primer sequences are shown in Table S2.

### 2.8. Western Blot Analysis

The IEC-6 cells were collected with cold PBS and extracted using RIPA buffer containing PMSF and phosphatase inhibitors. The protein concentrations were quantified with a BCA assay kit according to the manufacturer’s instructions. Equal protein amounts were electrophoresed on SDS-PAGE gels, transferred to a polyvinylidene fluoride membrane, and blocked with 5% milk. After incubation with antibodies, immunoreactivity was detected using ECL reagents (PerkinElmer, Waltham, MA, USA). The data were analyzed with the associated Image Lab, with n = 3 in each group.

### 2.9. Immunofluorescence Detection of γH2AX

The IEC-6 cells were washed with PBS twice and then blocked with 10% normal donkey serum containing 0.3% Triton-X-100. After incubation with the Phospho-Histone H2AX (Ser139) rabbit monoclonal antibody overnight at 4 °C, the cells were washed with PBS and incubated in the secondary Alexa Fluor 488 goat anti-rabbit antibody for 2 h at room temperature. Before taking images, cells were washed again with PBS and mounted with DAPI for 10 min. Fluorescent images were observed with a confocal laser scanning microscope (CLSM, LSM 700, Zeiss, Oberkochen, Germany) and processed using the ZEN imaging software.

### 2.10. Comet Assay

The comet assay (single cell gel electrophoresis) was performed with the Trevigen Comet AssayTM kit (Trevigen) according to the manufacturer’s protocol. Briefly, IEC-6 cells were collected in PBS to a concentration of 1 × 10^5^ cells/mL, mixed with 37 °C 1% LMA garose (low-melting agarose) and loaded on 2-well CometSlides. CometSlides were placed in the pre-cold lysis solution at 4 °C for 60 min, and then incubated in alkaline unwinding solution at room temperature for 20 min in the dark. CometSlides were transferred to a pre-cold fresh alkaline electrophoresis solution and subjected to electrophoresis using the CometAssay Electrophoresis System II (Trevigen) for 30 min (21 V). Slides were washed twice in dH_2_O for 5 min and 70% ethanol for 5 min. DNA was stained with 50 μL DAPI in a light-protected setting for 30 min and visualized using the confocal laser scanning microscope (CLSM, Carl Zeiss LSM 700).

### 2.11. Statistical Analysis

Metabolites were considered statistically significant when the *p*-value was <0.05 and VIP > 1. The MetaboAnalyst online software was used for multivariate analysis. For multivariate analysis, the following parameters were used: remove features with >80% missing values, exclude variables with missing values, mean intensity value, normalization by median, log data transformation, and Pareto data scaling. All values in the text and figures were expressed as mean ± SEM. Statistical analysis was performed using Prism v8.0 (GraphPad Software, La Jolla, CA, USA). A two-tailed Student’s *t*-test for comparison between two groups was used: * *p* < 0.05, ** *p* < 0.01, *** *p* < 0.001, ns not significant vs. control group. *p*-values less than 0.05 and 0.01 were regarded as significant and very significant, respectively.

## 3. Results

### 3.1. Basic Characteristics of the Participants

In this study, 77 participants (31 with UC and 46 healthy) were recruited to discover and evaluate their biomarkers. The demographic and general clinical characteristics of participants are summarized in Table S1.

### 3.2. Multivariate Statistical Analysis of Potential Biomarkers for UC

**Figure 1:**
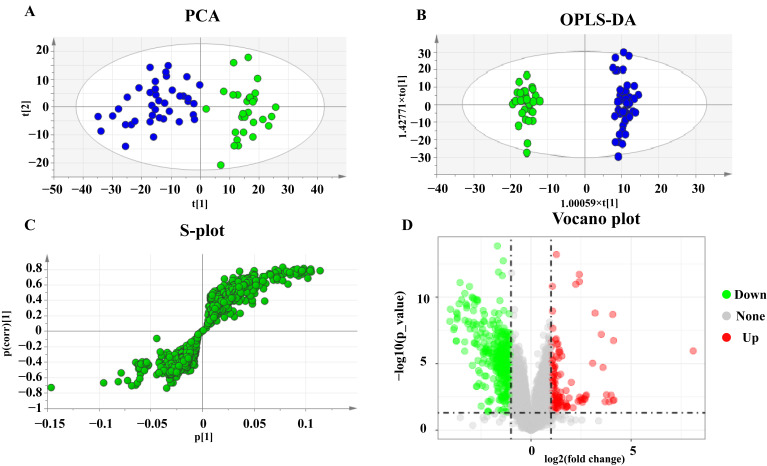
PCA, OPLS-DA score plots, and S-plots of healthy and UC groups based on HPLC-Q-TOF serum analysis. (**A**) PCA score plots of healthy and UC group. (**B**) OPLS-DA score plots of healthy and UC group. Blue represents the healthy group and green represents the UC group. (**C**) S-plot of healthy and UC group. (**D**) Volcano plot of healthy and UC group.

**Figure 2:**
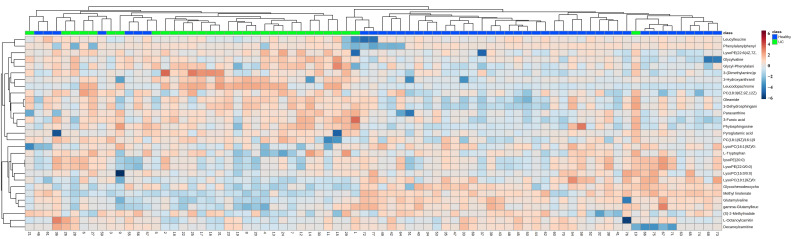
Heatmap of the 31 differential endogenous serum metabolites in the healthy and UC groups. Red represents the metabolites in high abundance and blue represents the metabolites in low abundance.

All observations acquired from the serum were analyzed using SIMCA-P software for multivariate statistical analysis. Principal component analysis (PCA) was used to perform unsupervised data analysis on the UC and control groups in order to reflect the inter-group and intra-group variability as a whole. As shown in Figure 1A, the two groups could be easily distinguished from each other, which showed that the difference of metabolic spectra between two groups was remarkable. Orthogonal partial least-squares discrimination analysis (OPLS-DA) is a supervised discriminant analysis used to predict the category of a sample. It can be seen from Figure 1B that there is an obvious trend of separation among the comparison groups (R^2^Y = 0.985, Q^2^ = 0.897). The variable importance for the projection (VIP) was calculated to measure the influence intensity and explanatory ability of the expression pattern of each metabolite on the classification and discrimination of each sample to assist in the screening of marker metabolites. After excluding exogenous metabolites and standardizing the data on the MetaboAnalyst 5.0 website, metabolites with VIP values over 1 and *p*-values acquired through the two-tailed Student’s *t*-test lower than 0.05 were considered as potential biomarkers, as listed in Table 2. A hierarchical clustering heatmap was used to present the data more intuitively (Figure 2).

### 3.3. Enrichment Analysis of Metabolic Pathway and Regulatory Enzymes

**Figure 3:**
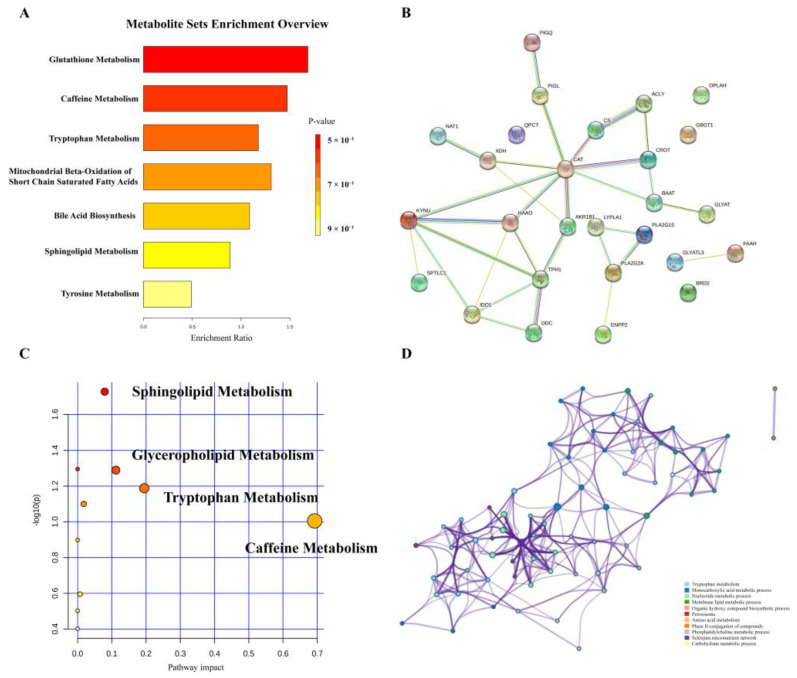
Enrichment analysis of metabolic pathways. (**A**) Overview of pathways related to the differential endogenous metabolites. (**B**) A summary of pathway analysis with Metascape. (**C**) Regulatory protein network map. (**D**) Regulatory enzyme GO enrichment analysis results.

To reveal the pathways of metabolites and their metabolic processes, significant metabolites were introduced into MetaboAnalyst 5.0 (https://www.metaboanalyst.ca/) (accessed on 2 September 2021) for pathway enrichment analysis. As shown in Figure 3A,B, the most affected pathways in patients with UC were glutathione metabolism, caffeine metabolism, tryptophan metabolism, mitochondrial beta-oxidation of short-chain saturated fatty acids, bile acid biosynthesis, sphingolipid metabolism, and tyrosine metabolism. Furthermore, we analyzed the pathway and enrichment analysis of related regulatory enzymes through STRING (https://string-db.org/) (accessed on 28 September 2021) and Metascape (https://www.metascape.org/) (accessed on 30 September 2021). The results of the protein interaction network and GO enrichment analyses showed that the function of regulatory enzymes mainly included tryptophan metabolism, monocarboxylic acid metabolic processes, nucleotide metabolic processes, and membrane lipid metabolic processes.

### 3.4. Validation of Potential Pro-Inflammatory and DNA Damage Activity of Pyroglutamic Acid

Among the differential markers we screened, pyroglutamic acid attracted our attention as it has been reported to be closely related to the development of a variety of tumors. As shown in Figure S3, its receiver operating characteristic (ROC) area under the curve (AUC) is 0.67, which means that pyroglutamic acid could be used as a biomarker for UC. As shown in Figure S4A, the statistics on the relative abundance of pyroglutamate found that 74.2% (23/31) of UC patients had values higher than the 95% confidence interval of healthy controls, and 32.3% (10/31) were more than 1.5-times higher than the 95% confidence interval. Using the modified Mayo score to classify UC patients into mild and moderate, we found that the relative abundance of pyroglutamic acid was not correlated with the severity of UC, and there was no significant difference between the relative abundance of mild and moderate UC patients (Figure S4B). Compared with UC patients diagnosed for less than 1 year, patients who had been diagnosed for 1–5 years or more than 5 years had higher pyroglutamic acid levels (Figure S4C). Thus, the pyroglutamic acid level was significantly upregulated with the duration of the disease. To explore whether pyroglutamic acid has the ability to promote the transformation of inflammation to intestinal cancer, intestinal epithelial IEC-6 cells were used to verify its potential pro-inflammatory and carcinogenic effects.

**Figure 4:**
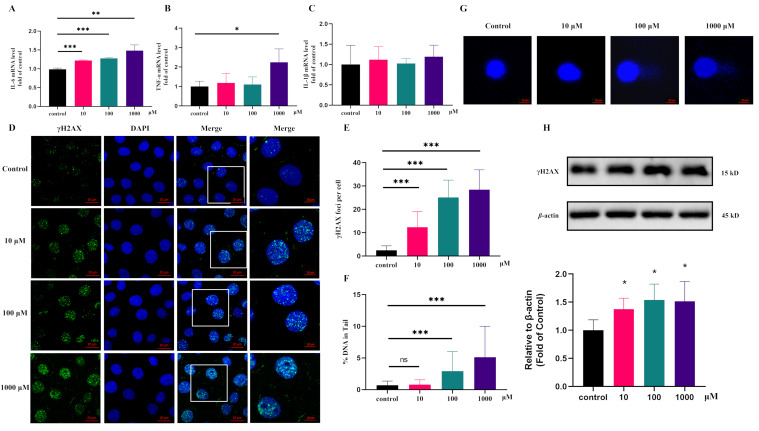
Pyroglutamic acid could promote inflammation and increase DNA damage in IEC-6 cells. (**A**) The mRNA levels of IL-6 detected by QPCR. (**B**) The mRNA levels of TNF-*α* detected by QPCR. (**C**) The mRNA levels of IL-1*β* detected by QPCR. (**D**) Representative images of γH2AX immunofluorescence staining. Image sizes are annotated by the scale bar in the lower right corner of the respective image. (**E**) Immunofluorescence histograms showing γH2AX foci per cell. (**F**,**G**) Comet assay and histograms showing %DNA in the tail. (**H**) Western blot of γH2AX. Data are presented as the means ± SEM. * *p* < 0.05, ** *p* < 0.01, *** *p* < 0.001, ns not significant vs. control group.

First, the mRNA levels of TNF-*α*, IL-1*β*, and IL-6 were detected, as these have been reported to be closely related to UC. Our results revealed that 10 μM pyroglutamic acid treatment for 24 h could significantly upregulate the IL-6 mRNA level in IEC-6 cells (Figure 4A,C). To explore the carcinogenic activity, we tested the effect of pyroglutamic acid on DNA damage. Pyroglutamic acid increased phosphorylation of histone H2AX (γH2AX), a surrogate marker for DNA damage in IEC-6 cells compared with untreated cells (Figure 4H). The immunofluorescence detection of γH2AX showed that 10 μM pyroglutamic acid could increase the number of γH2AX foci (Figure 4D,E). Moreover, measuring DNA damage via the comet assay showed that 100 μM pyroglutamic acid could increase the percentage of DNA in the tail (Figure 4F,G). These results all revealed that pyroglutamic acid could increase DNA damage and that it shows potential carcinogenic activity.

## 4. Discussion

Ulcerative colitis is a chronic, relapsing illness affecting millions of patients worldwide. However, the etiology and exact pathophysiology of UC remain unknown. A delayed diagnosis imposes serious clinical implications [19]. In this study, changes in overall serum metabolism were revealed by comparing ulcerative colitis to healthy controls. A total of 31 metabolites significantly varied between the two groups, including indoles, sphingomyelin, bile acids, and amino acids.

In this study, several metabolites of the indole family and pathway were altered in patents with UC, such as leucodopachrome and L-tryptophan. Indole derivatives are important ligands for the aryl hydrocarbon receptor (AhR). AhR is involved in intestinal mucosal homeostasis by acting on innate and adaptive immune cells, as well as epithelial renewal and mucosal barrier function [20,21]. Tryptophan plays a central role in AhR activation and can be metabolized by the intestinal flora, which is essential for maintaining the immune balance of the mammalian intestinal tract [22,23]. Tryptophan added in food could reduce DSS-induced colitis through aryl hydrocarbon receptors in mice [24,25]. At the same time, it is also a biomarker for colon cancer [22]. We found that tryptophan was significantly reduced in the serum of patients, which may be related to intestinal dysbiosis in UC patients.

Sphingolipids such as 3-dehydrosphinganine and phytosphingosine showed increased concentrations in this study. The role of sphingomyelin components in UC is not fully understood. Increased sphingomyelin concentrations in both animal models of colitis and patients with IBD suggest that sphingomyelin has a part in chronic intestinal inflammation [26,27,28].

We also found that bile acids were altered in UC patients, such as glycocholic and glycochenodeoxycholate. Bile acids have many physiological roles, including glucose regulation and intestinal motility. The processes by which bile acids exert their multiple roles are complex and dependent on the host and gut bacteria [29,30,31]. The genes required to convert primary bile acids into secondary bile acids were reduced in UC patients, as well as secondary bile acid-producing bacteria. Secondary bile acid supplementation alleviated intestinal inflammation through the TGR5 bile acid receptor in a mouse model of colitis [32]. A detrimental feedback loop could be created where inflammation results in reduced bile absorption, and therefore increased bile in the lumen, which in turn causes increased inflammation.

Our study indicated that multiple amino acids were increased in the serum of UC patients, such as glycylvaline. The high number of amino acids in active UC is likely due to malabsorption [33]. These results suggest that amino acid balance should be stressed as an important area for patients with an active disease as well as those in remission [34].

Another amino acid involved in glutathione metabolism, pyroglutamic acid, attracted our attention. Pyroglutamic acid, also known as 5-oxo-proline, is the cyclic lactam in glutamic acid [35,36]. Glutathione metabolism participates in redox reactions and energy metabolism. Reactive oxygen species have been shown to contribute to tissue damage in patients with ulcerative colitis and Crohn’s disease [37,38]. In this study, pyroglutamic acid has a high contribution rate to the metabolomic pilot of UC, and it was found to be significantly upregulated in the serum of UC patients for the first time. Pyroglutamic acid has been reported to be upregulated in the stool of IBS patients, which is related to Lactobacillus [39]. One study showed that, compared with a control group, the level of pyroglutamic acid is significantly higher in UC-model rats [40]. More importantly, it is also a biomarker for a variety of cancers. The levels of pyroglutamic acid in the serum of patients with cervical cancer, gastric cancer, and breast cancer are significantly increased [41,42,43,44,45]. In our study, the statistics on the relative abundance of pyroglutamate showed that 74.2% (23/31) of UC patients had values higher than the 95% confidence interval of healthy controls, and 32.3% (10/31) had values more than 1.5-times higher than the 95% confidence interval. Compared with UC patients diagnosed for less than 1 year, patients who had been diagnosed for 1–5 years or more than 5 years had higher pyroglutamic acid levels, which means that pyroglutamic acid is correlated with the duration of the disease. This suggests that pyroglutamic acid could not only be a biomarker for distinguishing UC status, but that it is also an active metabolite that promotes the transformation of UC to CRC. However, the effects of pyroglutamic acid in vivo and in vitro have not been well studied.

Pyroglutamic acid is naturally present in mammalian tissues and fluids, and the content in the serum of a healthy person could reach the micromolar level [46]. Our results showed that 10 μM of pyroglutamic acid could upregulate the level of inflammatory factors in IEC-6 cells and cause the upregulation of phosphorylation of H2AX, an early indicator of DNA damage [47]. DNA damage blocks the cell cycle and quickly activates the repair mechanism to prevent errors from being passed on to future generations. However, repeated damage repair increases the probability of DNA mutations, causing genome instability, and the accumulation of mutations increases the possibility of cancer [48,49,50,51,52]. In order to find out whether the upregulation of pyroglutamic acid in the disease state is related to the intestinal flora, we cultured patients’ intestinal flora to detect the relative content of pyroglutamic acid and found that the flora does not metabolize pyroglutamate acid. This result suggests that the upregulation of pyroglutamic acid may be related to its metabolic enzymes, such as QPCT and OPLAH, which needs to be further verified in vivo and in vitro.

## 5. Conclusions

In summary, this study analyzed the metabolic profiles of UC samples and found potential biomarkers. Our results have revealed that pyroglutamic acid is a unique metabolite that was significantly increased in the serum of UC patients. Moreover, the role of pyroglutamic acid in UC was further analyzed and it was found that it upregulates inflammatory factors and increases DNA damage. This study contributes to a better understanding of this potential mechanism and proposes effective markers for the clinical diagnosis and treatment of UC.

## Figures and Tables

**Table 1:** The relative standard deviation (RSD%) of retention time and peak area in QC samples.

*m*/*z*	RSD of Retention Time (%)	RSD of Peak Area (%)
496.1399	0.0088	7.8288
524.3700	0.0829	5.5716
100.0762	0.0698	4.4323

**Table 2:** Key differential metabolites between the healthy and UC groups.

Metabolites	*m*/*z*	Rt (min)	FC	*p*-Value	VIP	Metabolic Pathways	Enzymes Genes
3-Furoic acid	113.0181	2.0264	1.23	7.70 × 10^−4^	3.77		
Pyroglutamic acid	130.0465	2.0868	1.27	8.09 × 10^−3^	3.40	Glutathione metabolism, glutathione synthetase	QPCT, OPLAH
(*S*)-2-Methylmalate	149.0444	12.8028	0.65	3.56 × 10^−2^	1.99	Fatty acid metabolism, lipid metabolism	
3-Hydroxyanthranilic acid	154.0442	2.0607	12.09	1.93 × 10^−4^	2.77	Tryptophan metabolism	KYNU, HAAO, CAT
Glycylvaline	175.1094	2.1603	1.48	4.45 × 10^−^^3^	1.61		
Paraxanthine	181.0705	2.0425	1.40	4.93 × 10^−^^5^	1.33	Caffeine metabolism	NAT1, NAT2, XDH
Leucodopachrome	196.0561	8.1400	2.74	1.10 × 10^−^^6^	7.26	Tyrosine metabolism	
L-Tryptophan	205.0974	5.7118	0.83	1.45 × 10^−^^3^	9.65	Tryptophan metabolism	DDC, IDO1, TPH1
3-(Dimethylamino) propyl benzoate	208.1290	4.8659	6.81	2.255 × 10^−^^3^	1.82		
Glycyl-Phenylalanine	223.1056	5.2276	1.27	1.74 × 10^−^^2^	1.22		
Leucylleucine	245.1841	7.8416	0.66	3.56 × 10^−^^3^	2.36		
Glutamylvaline	247.1279	3.5562	0.37	2.39 × 10^−^^12^	3.35		
gamma-Glutamylleucine	261.1455	6.2629	0.57	8.60 × 10^−^^10^	2.86		
Oleamide	282.2768	25.5358	1.76	1.20 × 10^−4^	1.05	Fatty acid metabolism, lipid metabolism	FAAH, PLA2G2A
L-Octanoylcarnitine	288.2164	15.4359	0.69	2.51 × 10^−^^2^	1.68	Mitochondrial beta-oxidation of short-chain saturated fatty acids	CROT
Methyl linolenate	293.2493	42.8410	0.70	1.99 × 10^−^^8^	4.07		
17-Hydroxylinolenic acid	295.2280	36.7773	0.11	1.04 × 10^−^^8^	1.60	Fatty acid metabolism, lipid metabolism	
3-Dehydrosphinganine	300.2854	25.5358	1.78	6.96 × 10^−4^	2.16	Sphingolipid metabolism	GBGT1, PIGL, SPTLC1
Phenylalanyl phenylalanine	313.1578	10.2882	0.70	9.95 × 10^−^^3^	6.74		
Decanoylcarnitine	316.2458	19.5269	0.65	3.45 × 10^−^^2^	2.21	Fatty acid metabolism, lipid metabolism	
Phytosphingosine	318.3016	21.1592	1.33	7.22 × 10^−^^3^	4.95	Sphingolipid metabolism	GBGT1, PIGL, PIGQ
Glycocholic acid	448.3052	23.5106	0.16	5.79 × 10^−^^6^	1.08	Bile acid biosynthesis	BAAT, GLYAT, GLYATL3
Glycochenodeoxycholate	450.3251	25.9395	0.13	1.34 × 10^−^^5^	2.44	Bile acid biosynthesis	BAAT, GLYAT, GLYATL3
LysoPC(15:0/0:0)	482.3287	31.1631	0.68	2.90 × 10^−4^	2.16	Glycerophospholipid metabolism, lipid metabolism	LYPLA1, PLA2G15
LysoPC(16:1(9Z)/0:0)	494.3247	30.8185	0.49	7.76 × 10^−4^	1.28	Glycerophospholipid metabolism, lipid metabolism	LYPLA1, PLA2G15
LysoPE(20:0)	516.3382	16.7751	0.76	3.98 × 10^−^^3^	2.81	Fatty acid metabolism, lipid metabolism	ENPP2
LysoPC(18:1(9Z)/0:0)	522.3610	35.3163	0.30	4.35 × 10^−^^6^	5.12	Fatty acid metabolism, lipid metabolism	LYPLA1, PLA2G15
LysoPE(22:6(4Z,7Z,10Z,13Z,16Z,19Z)/0:0)	526.2905	31.5744	1.36	2.40 × 10^−^^3^	1.91	Glycerophospholipid metabolism, lipid metabolism	ENPP2
LysoPE(22:0/0:0)	560.3676	17.1108	0.75	2.84 × 10^−^^3^	3.11	Glycerophospholipid metabolism, lipid metabolism	ENPP2
PC(18:1(9Z)/16:1(9Z))	780.5490	51.6295	1.48	1.33 × 10^−^^2^	4.85	Phosphatidylcholine biosynthesis	LYPLA1, PLA2G15
PC(18:3(6Z,9Z,12Z)/18:1(9Z))	782.5640	51.6295	1.23	3.16 × 10^−^^2^	2.69	Phosphatidylcholine biosynthesis	LYPLA1,PLA2G15

## Data Availability

The data presented in this study are available in article and supplementary material.

## Supplementary Materials

The following supporting information can be downloaded at: https://www.mdpi.com/article/10.3390/metabo12100997/s1, Figure S1: PCA scores plot of tested samples and QC samples; Figure S2: The total ion chromatograms (TICs) of the QC samples of serum; Figure S3: ROC curve analysis of pyroglutamic acid in the discovery set; Figure S4: Statistics of relative abundance of pyroglutamic acid between the healthy and UC group in serum; Table S1: Clinical characteristics of the subjects; Table S2: The primer sequence of IL-6, IL-1*β*, TNF-*α* and *β*-actin.
