# Supplementary material for: Discovery and Validation of Potential Serum Biomarkers with Pro-Inflammatory and DNA Damage Activities in Ulcerative Colitis: A Comprehensive Untargeted Metabolomic Study
Source: Metabolites. 2022 Oct 20;12(10):997. doi: 10.3390/metabo12100997 (PMC9609580; doi:10.3390/metabo12100997)
Supplement: Supplementary file 1 [file metabolites-12-00997-s001.zip › metabolites-1966870-supplementary.pdf]

Supporting Information for

Discovery and validation of potential serum biomarkers with pro-inflammatory and DNA damage activities in ulcerative colitis: A comprehensive untargeted metabolomic study

Mingxiao Li[1], Rui Zhang[1], Mingjie Xin[1], Yi Xu[2], Shijia Liu[2], Boyang Yu[1], Boli Zhang[3*] and Jihua Liu[1*]

[1] Jiangsu Provincial Key Laboratory for TCM Evaluation and Translational Development, School of Traditional Chinese Pharmacy, China Pharmaceutical University, Nanjing 211198, China
[2] Department of Pharmacy, Jiangsu Province Hospital of Chinese Medicine, Affiliated Hospital of Nanjing University of Chinese Medicine, Nanjing 210029, China
[3] State Key Laboratory of Modern Chinese Medicine, Tianjin University of Traditional Chinese Medicine, Tianjin 301617, China

Table of contents

Figure S1. PCA scores plot of tested samples and QC samples
Figure S2. The total ion chromatograms (TICs) of the QC samples of serum
Figure S3. ROC curve analysis of pyroglutamic acid in the discovery set
Figure S4. Statistics of relative abundance of pyroglutamic acid between the healthy and UC group in serum
Table S1. Clinical characteristics of the subjects
Table S2. The primer sequence of IL-6, IL-1β, TNF-α and β-actin

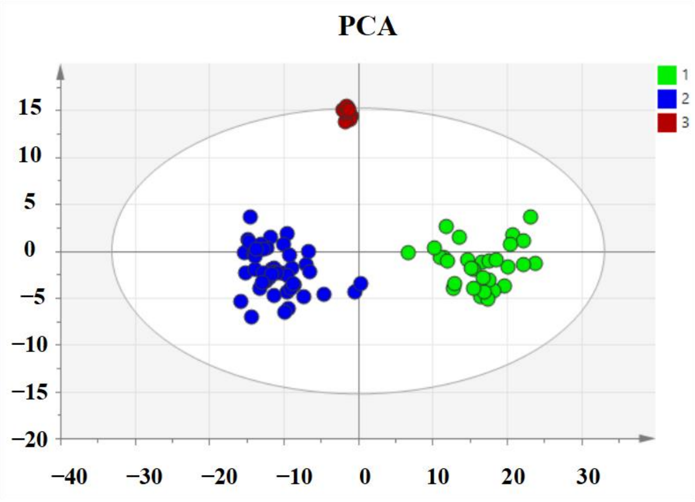

Figure S1. PCA scores plot of tested samples and QC samples. Blue points represent healthy group and green points represent UC group, red points represent QC samples.

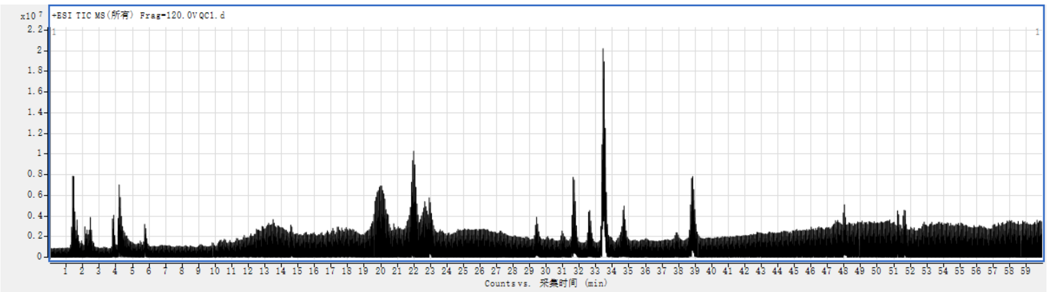

Figure S2. The total ion chromatograms (TICs) of the QC samples of serum.

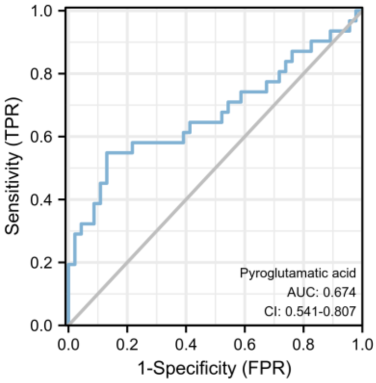

Figure S3. ROC curve analysis of pyroglutamic acid in the discovery set. AUC: area under the curve.

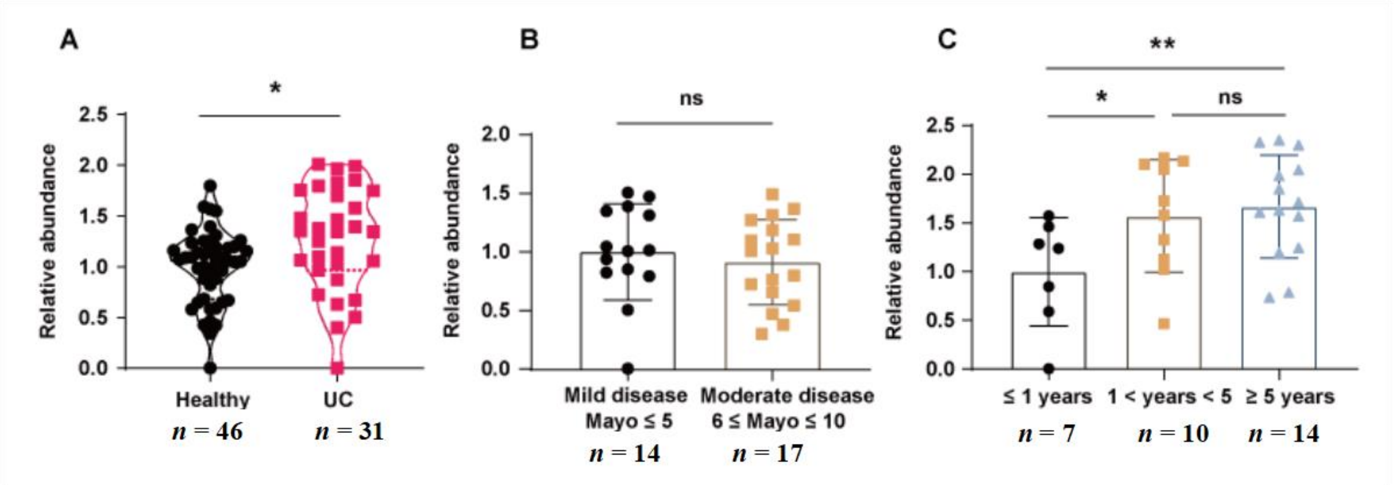

Figure S4. Statistics of relative abundance of pyroglutamic acid between the healthy and UC group in serum.(A) Relative abundance of pyroglutamic acid between the healthy and UC group in serum. (B) Relative abundance of pyroglutamic acid accoarding to the Mayo scores in UC patients. (C) Relative abundance of pyroglutamic acid accoarding to the duration in UC patients. Data are presented as the means ± SEM. * $p < 0.05$, ** $p < 0.01$, *** $p < 0.001$, ns not significant vs. healthy group.

Table S1. Clinical characteristics of the subjects.

Characteristics	Healthy	UC
Number	46	31
Male	10	10
Female	36	21
Age (years), mean ± SD	30.48 ± 6.38	37.00 ± 9.44
UC history (years)		4.81(0, 20)
Mayo score		5.45 ± 2.05

Table S2. The primer sequence of IL-6, IL-1*β*, TNF-*α* and *β*-actin.

Name	Primer (5'-3')
F(IL-6)	AGTTGCCTTCTTGGGACTGA
R(IL-6)	ACTGGTCTGTTGTGGGTGGT
F(IL-1*β*)	AGAGTGTGGATCCCAAACAA
R(IL-1*β*)	AGTCAACTATGTCCCGACCA
F(TNF-*α*)	TCTTCTGTCTACTGAACTTCGGGG
R(TNF-*α*)	ATGGAACTGATGAGAGGGAGCC
F(*β*-actin)	GAGACCTTCAACACCCC
R(*β*-actin)	ATAGCTCTTCTCCAGGGAGG
